# Health-related quality of life, needs, and concerns among cancer survivors referred to rehabilitation in primary healthcare setting

**DOI:** 10.2340/1651-226X.2024.19636

**Published:** 2024-03-14

**Authors:** Mette T. Sandager, Sine Rossen, Dorte T. Hofland, Claus V. Nielsen, Thomas Maribo

**Affiliations:** a Copenhagen Centre for Cancer and Health, Municipality of Copenhagen, Copenhagen, Denmark; b Department of Public Health, Aarhus University, Aarhus, Denmark; c DEFACTUM Central Denmark Region, Aarhus, Denmark; d Social Medicine and Rehabilitation, Region Hospital Goedstrup, Denmark

**Keywords:** Cancer rehabilitation, health-related quality of life, patient-reported outcomes, functional assessment of cancer therapy-general, International Classification of Functioning, Disability and Health, holistic rehabilitation

## Abstract

**Background and purpose:**

There is a growing need for rehabilitation services beyond hospitals. This study aims to describe challenges faced by cancer survivors (CSs) referred for rehabilitation in primary healthcare, employing standardized scales measuring health-related quality of life (HRQOL) and open-ended questions. Furthermore, the study explores the applicability of patient-reported outcomes (PROs) in comprehensively understanding challenges encountered by CSs.

**Material and methods:**

This cross-sectional study involves CSs referred for cancer rehabilitation in a primary healthcare setting, including those participating in PROs as a part of routine practice. HRQOL was assessed using the Functional Assessment of Cancer Therapy-General (FACT-G). The International Classification of Functioning, Disability and Health (ICF) framed the analysis of responses to open-ended questions ‘what concerns you the most?’ and ‘what matters to you?’

**Results:**

FACT-G showed the lowest scores for functional well-being (14.4) and emotional well-being (16.6), with higher scores for physical well-being (18.9) and social/family well-being (21.1). Responses to open-ended questions unveiled worries about everyday life and how cancer will impact family well-being presently and in the future. Furthermore, CSs reported a need to maintain normality and proactively address the challenges posed by the disease.

**Interpretation:**

CSs referred for rehabilitation in primary healthcare experience comprehensive challenges necessitating a holistic rehabilitation approach. This includes interventions supporting CSs in dealing with uncertainty, regaining a sense of control, and addressing family well-being concerns. When using PROs for need assessment, the combination of validated HRQOL scales and open-ended questions is crucial for an in-depth understanding of CSs’ challenges.

## Introduction

An increasing number of persons are living with and beyond cancer [1, 2]. Receiving a cancer diagnosis and undergoing cancer treatment can leave patients with challenges that can have a negative impact on functioning and health-related quality of life (HRQOL) [3–5]. Rehabilitation of cancer survivors (CSs) has the potential to improve functioning and quality of life [5–7]. Rehabilitation is defined as ‘a set of interventions designed to optimize functioning and reduce disability in individuals with health conditions in interaction with their environment’ [8]. Rehabilitation focuses on reaching and maintaining optimal physical, sensory, intellectual, psychological and social function and improve quality of life.

Specialized problems faced by CSs should be managed within a hospital setting (e.g. lymphedema treatment). Other more general and non-diagnosis specific problems (e.g. physical training) can be handled in a primary healthcare setting. Outpatient cancer rehabilitation has been shown to improve physical and psychosocial functioning [7]. Increased cancer incidence and improved survival have brought attention to the need for general rehabilitation in primary healthcare setting. Nevertheless, a substantial proportion of persons with cancer still report unmet needs for general rehabilitation [9–11].

Comprehensive understanding of rehabilitation needs is essential to ensure that rehabilitation services align with the complex requirements of persons living with cancer. However, a knowledge gap persists concerning the rehabilitation needs of CSs after hospitalization [6]. Although most rehabilitation in primary health care settings is independent of the specific cancer diagnosis, there are only a few studies on cancer rehabilitation including CSs with different cancer diagnoses [12, 13], and none of them focus on an outside hospital setting.

Patient needs can be systematically assessed by validated patient‐reported outcome (PRO) instruments. PRO is patient’s own report about their health status without interpretation of others. PRO includes validated questionnaires that measure disease‐specific health issues, HRQOL etc. [14]. Analysing PRO on a population‐level holds the potential to identify gaps in CSs’ needs and services and thereby guide policy decisions in relation to service delivery [15]. However, no instruments can be expected to capture all aspects experienced by CSs, and we hypothesize that a more comprehensive assessment of needs can be achieved by combining HRQOL measures with open-ended questions.

This study aims to thoroughly describe the challenges of CSs referred to rehabilitation in a primary healthcare setting. It is our hypothesis that combining standardized scales of HRQOL with responses from open-ended questions will reveal new nuances and a deeper understanding of CSs’ rehabilitation needs outside the hospital setting.

## Material and methods

### Study design

This cross-sectional study uses data collected as part of routine rehabilitation in a primary healthcare setting.

### Study setting and participants

Denmark is administratively and geographically divided into municipalities responsible for delivering welfare services including general rehabilitation. The municipalities provide general rehabilitation for persons with cancer within primary healthcare settings, while hospitals are responsible for highly specialized rehabilitation [16]. According to Danish health legislation, all CSs should receive an individual assessment of rehabilitation needs by a doctor before being discharged from regular cancer treatment at the hospital. If it is assessed that the patient requires rehabilitation, a referral for rehabilitation in the municipality will be sent, and the patient will be invited for a need assessment interview, where a rehabilitation plan is made in cooperation with the patient. Almost all municipalities offer rehabilitation for CSs with some variation in capacity and services offered [17].

The study took place at Copenhagen Centre for Cancer and Health (CCCH). This primary healthcare centre is responsible for rehabilitation of cancer CSs living in Copenhagen municipality (population of around 650,000 inhabitants). Yearly, around 1,200 patients are referred to rehabilitation at CCCH, which is around 45% of newly diagnosed patients in the municipality.

PROs are used as a tool for individual need assessment and to evaluate the general health status of the referred CSs. We included all CSs booked for an initial needs assessment interview from 1 April 2019 to 31 August 2022 who had filled out the PRO. Sixteen per cent were not included because they were non-Danish speaking or because they were in such a condition that the health professional that contacted the CS considered it unethical due to, for example, cognitive impairment.

### Data collection

The PRO was filled out at home using a smartphone, tablet, or computer approximately 1 week prior to the initial interview. If unable to fill out at home, it was possible to fill out the PRO on a tablet when arriving for the initial interview. The PRO consists of 62 items and is a combination of validated questions and scales (Functional Assessment of Cancer Therapy-General [FACT-G]), self-constructed items and open-ended questions.

The core of the PRO is the FACT-G. The FACT-G is a 27-item questionnaire designed to measure cancer-specific HRQOL in CSs. The reliability and validity of FACT-G are well established [18]. In FACT-G items are rated on a 5-point Likert scale ranging from 0 (not at all) to 4 (very much).

The FACT-G comprises four subscales: physical well-being, social/family well-being, emotional well-being, and functional well-being. An overall HRQOL score (total FACT-G score) is obtained by averaging the item scores, with higher scores indicating better HRQOL. The FACT-G scores were calculated according to the Functional Assessment of Chronic Illness Therapy website scoring algorithm [19]. Minimal important differences have been estimated to 5–6 points for total FACT-G, and 2–3 points for the subscales [20].

Qualitative comments were obtained from the open-ended questions ‘what concerns you the most?’ and ‘what matters to you?’. The open-ended questions are placed as the last questions in the PRO questionnaire.

Information on cancer diagnosis was retrieved from the patient journal. All other demographic (gender, age, education) and disease-related (time since diagnosis and recurrence of cancer) variables are self-reported.

The use of data from patient records was approved by the regional ethical committee (Nr. R-22060676).

## Data analysis

Data was analysed using both FACT-G and open-ended questions to obtain a comprehensive understanding of the challenges that CSs faces.

### Data analysis of HRQOL

Descriptive statistics (numbers and proportions) were used to examine HRQOL for overall FACT-G score and FACT subscale scores.

### Data analysis of comments from open-ended questions

The International Classification of Functioning, Disability and Health (ICF) was used for coding the answers to the open-ended questions. The ICF classifies functioning and disability according to the components Body Functions, Body Structures, Activity and Participation, and Environmental Factors. The classification encompasses more than 1,500 categories [21]. In recent years, ICF has increasingly become the common language when describing functioning and disability in rehabilitation [22] and there are prior examples of ICF successfully being used as a coding instrument [23]. Comments were extracted and linked according to the ICF linking rules [24].

To limit the scope, only answers from 20 CSs were analysed for each cancer diagnosis groups. CSs with unknown cancer diagnosis group were analysed as a group. The answers were randomly chosen via computer generation. For each answer, main concepts and additional concepts were identified. Subsequently, the identified concepts were linked to an ICF category. Main concepts were categorized as ‘not definable’ if there was not enough information to select the most precise ICF category and as ‘not covered’ if it was not included in the ICF universe.

Early in the process, it was discovered that the answers to the two questions ‘what concerns you the most?’ and ‘what matters to you?’ were generally understood to be the same. Consequently, the two open-ended questions were merged.

The focus of ICF is the present level of functioning. Several comments in the material concerned concerns about something that might happen in the future. In these cases, ICF category b152 (emotional functions) was given as additional category.

An example of the ICF linking process is presented in Table 1.

**Table 1:** Example of ICF linking process.

Comment	Main concept	Add. concept	Main ICF category	Add. ICF category
‘Will I be able to be there for my family in the future?’ ’To feel energetic’	Family To have energy	Worries about the future	e310 (immediate family) b130 (energy and drive functions)	b152 (emotional functions)

ICF: International Classification of Functioning, Disability and Health.

## Results

Overall response rate for CSs receiving a PRO was 89.4%. Generally, there was a lower response among persons over 75 years old and those with shorter education (data not shown).

Of the initial sample of 3,018 CSs, 2,643 (87.6%) answered the FACT-G and were available for quantitative analysis of HRQOL.

Patient characteristics are presented in Table 2.

**Table 2:** Demographic and cancer characteristics of persons included.

Characteristic		*N*	%
	Sample size	2,643	100
Age (years)	≤39	271	10.3
	40–64	1,315	49.8
	65–74	717	27.1
	≥75	340	12.9
Gender	Female	1,711	64.7
	Male	932	35.3
Educational level	Mandatory school	295	11.2
	Senior high school or vocational education	501	19.0
	Short higher education	289	10.9
	Intermediate higher education	824	31.2
	Higher education	661	25.0
	Missing	73	2.8
Cancer diagnosis (ICD code)	Breast (C50)	848	32.1
	Digestive organs (C15–C26)	439	16.6
	Respiratory and intrathoracic organs (C30–C39)	273	10.3
	Male genital organs (C60–C63)	197	7.5
	Lymphoid tissue (C81–C90)	173	7.5
	Female genital organs (C51–C58)	145	5.5
	Lip, oral cavity, and pharynx (C00–14)	143	5.4
	Ill-defined, secondary, and unspecified sites (C76–C80)	65	2.5
	Urinary tract (C64–C68)	60	2.3
	Haematopoietic tissue (C91–C96)	59	2.2
	Eye, brain, and other parts of central nervous system (C69–C72)	44	1.7
	Melanoma and other malignant neoplasms of skin (C43–C44)	37	1.4
	Mesothelial and soft tissue (C45–C49)	26	1.0
	Unknown	134	5.1
Reccurrence of cancer	New cancer (not reccurrence)	2,100	79.5
	Recurrence of cancer	354	13.4
	Missing	189	7.2
Time since cancer diagnosis	0–3 months	1,536	58.1
	4–11 months	644	24.4
	1–3 year	248	9.5
	More than 3 years	172	6.5
	Missing	43	1.6

### FACT-G scores

In Table 3, HRQOL is presented as total FACT-G score and FACT-G subscale scores.

**Table 3:** Mean total FACT-G scores and subscale scores for patients with cancer.

Scale	Number of items	Score range	Mean (SD)
Total FACT-G	27	0–108	71.1 (15.9)
Physical well-being	7	0–28	18.9 (5.5)
Emotional well-being	6	0–28	16.6 (4.6)
Functional well-being	7	0–28	14.4 (5.6)
Social/family well-being	7	0–24	21.1 (5.2)

FACT-G: functional assessment of cancer therapy-general.

Mean total FACT-G score for all CSs just prior to starting rehabilitation was 71.1.

The FACT-G subscales revealed lowest scores for functional well-being (14.4) and emotional well-being (16.6) and higher scores for physical (18.9) and social/family well-being (21.1).

FACT-G individual item scores are presented in Appendix 1, and differences in total FACT-G scores between cancer diagnosis groups are presented in Appendix 2.

### Needs and concerns

A total of 2,363 out of the 2,646 (89%) individuals who completed the PRO provided qualitative comments in response to the open-ended question ‘What concerns you the most?’ and/or ‘What matters to you’.

Twenty CSs were randomly selected from each cancer diagnosis groups, resulting in a total of 280 CSs included in this analysis.

The total number of meaningful concepts linked to ICF components (activities and participation; body functions; body structures; environmental factors) and the number of meaningful concepts which could not be linked are presented in Table 4. In 57 cases, the comments for the two questions ‘what concerns you the most?’ and ‘what matters to you?’ related to the same ICF category and were only coded to count one time. Second level categories are presented if five or more comments are present.

**Table 4:** Qualitive comments categorized according to ICF components.

ICF component	Main concept % (*n*)	Main concept ICF categories (*n*)*	Add. Concept % (*n*)	Add. Concept ICF categories (*n*)*
Activities and participation	22 (181)	d570 – Looking after one’s health (37), d845 – Acquiring, keeping and terminating a job (28), d240 – Handling stress and other psychological demands (17), d920 – Recreation and leisure (17), d760 – Family relationships (12), d770 – Intimate relationships (9), d230 – Carrying out daily routine (7)	14 (26)	d240 – Handling stress and other psychological demands (14)
Body functions	15 (125)	b152 – Emotional functions (28), b130 – Energy and drive functions (22), b280 – Sensation of pain (14), b530 – Weight maintenance functions (8), b730 – Muscle power functions (7)	71 (127)	b152 – Emotional functions (118)
Body structures	1 (5)		2 (3)	
Environmental factors	18 (153)	e310 – Immediate family (110), e320 – Extended family (26)	5 (9)	
ND (Not defined)	10 (86)	Overall condition of the body (19), Overall health (16), Side effects/late effects (15), Independence (13), Stay active (8)	4 (8)	
NC (Not covered)	34 (282)	Disease free (67), normality (34), Death (32), Treatment (29), Recurrence (23), development of disease (22), Enjoy life (17), Good life/Quality of life (13), Status of disease (7)	4 (7)	
Total	100 (832)		100 (182)	

ICF: International Classification of Functioning, Disability and Health.

* ICF categories were n > 5 are presented.

A total of 832 main concepts were identified, of which 22% were related to activities and participation, 18% to environmental factors, 15% to body functions and 1% to body structures, 34% of the main concepts were categorized as not covered (NC) and 10% as not defined (ND).

A total of 182 additional concepts were identified. Most of the additional concepts are linked to the body function category (71%) with b152 (emotional functions) being the by far most dominant ICF category.

The most reported issue related to activity and participation were d570 – looking after one’s health, d845 – Acquiring, keeping, and terminating a job and d240 – Handling stress and other psychological demands. For environmental factors, the most frequently linked category was e310 – immediate family, which refer to the support that family provides as well as thoughts about how the cancer disease has an impact on children and family’s well-being.

For body function, mental functioning was more prominent than physical functioning. The by far most mentioned category was b152 – emotional functions. The majority of these were coded as additional category and related to worries for something that might happen in the future. b130 – energy and drive functions were another frequently reported category.

Examples of meaningful concepts which were categorized as ‘not covered’ include concerns of being disease-free, holding on to normality, thoughts about death, treatment and thoughts about recurrence and development of disease. More than half of NC categories relates to cancer-specific issues (treatment, recurrence, status of disease, etc.). Similarly, there are several statements that were not definable to ICF that related to side effects of treatment.

## Discussion

The primary aim of this study was to describe HRQOL, needs and concerns by combining validated measures of HRQOL and open-ended questions.

Results from this study indicate that CSs accessing rehabilitation in a primary healthcare setting experience comprehensive challenge, especially regarding functioning and emotional matters. Furthermore, answers to the open-ended questions revealed a large number of CSs reporting concerns about how their future every-day life will be, which functional limitations they might experience and what consequences this might have for themselves and others.

The mean total FACT-G score in this study were 71.1, which is lower than the observed in a normal population where a score of 86.5 has been found [25]. This difference exceeds the 5–6-point threshold considered a minimal important difference [20].

Interestingly, in relation to social/family well-being, the cancer population had higher score (21.1) than another non-cancer population (20.2). This also is seen in other studies [26]. Even though CSs had high scores in FACT-G social/family well-being, the social/family domain was still a prominent theme in the open-ended questions. The comments primarily relate to concerns about how the cancer diagnosis will affect the life and mental state of children, spouse, or other family members or what will happen to them if the patient does not survive the cancer. This is a nuance not covered by the scale, which solely focuses on how relatives can constitute a resource for the patient.

Knowledge emerging from the open-ended questions revealed a need for looking after one’s health, for example, by being physical active and eating a healthy diet. This is a nuance that is not covered by FACT-G. Cancer is a disease that is beyond the patient’s control, and this might be a strategy for coping with uncertainty. Engaging in healthy behaviours and activities has shown to be a way of strengthening a sense of control [27, 28], and the results reveal a need to have a focus on such interventions in cancer rehabilitation.

Cancer rehabilitation typically includes interventions such as physical therapy and activity, psychological interventions, and guidance on economic and work-related issues [3, 7]. The results of this study call for a focus that addresses wider aspects of functioning, including initiatives to address the stress that comes with dealing with uncertainty and interventions that empower CSs to have a greater sense of control over their illness and treatment.

Furthermore, CSs expressed many thoughts and concerns that related specifically to the cancer disease and treatment. This points to the fact that health professionals working with cancer rehabilitation need to possess a certain level of cancer-specific knowledge; alternatively, involvement of the specialist level at the hospital should be maintained in some form.

Hence, to address the rehabilitation needs of CSs, there is a need for a holistic rehabilitation approach that, in addition to physical, psychological, work-related interventions, encompasses a focus on helping CSs to manage uncertainty about the future and regaining a sense of normality, handling concerns about family’s wellbeing and future everyday life, and helping CSs to regain a sense of control by taking active steps against the disease.

In recent years, there has been an increased focus on integrating PRO as a part of routine patient care and as an instrument of individual need-assessment in the clinical consultation [29]. The use of PRO has been demonstrated to improve communication between patients and professionals and help to identify patient needs [30, 31].

There are a variety of PROs available, and validated scales are the core of most PROs. Our results, however, showed that open-ended questions are adding important dimensions to the understanding of the patient’s response that cannot be extrapolated from validated scales of HRQOL. A response rate on 89% to the open-ended questions also indicates that patients often have more to say than the validated scales capture.

At an organizational or system level, it is important to choose instruments that can be used to generalize or compare across different groups or settings. But, at an individual level, especially when the purpose is thorough need assessment and clinical decision making, this study emphasises the importance of including open-ended questions in addition to the validated scales. Open-ended questions were essential in obtaining an in-depth understanding of the individual patient’s unique challenges. The inability of validated instruments to fully assess functioning [32, 33] and the importance of adding open-ended questions [34] have been identified by others.

ICF is increasingly being recognized as the standard language to describe and measure functioning [22]. To a large extent, ICF proved suitable for describing functioning for CSs. But this study exposed some challenges within the ICF framework in capturing some important elements of CSs’ self-reported needs and concerns. ICF focuses on what have manifested itself in a person’s functioning. An interesting finding was that CSs are strongly affected of the uncertain situation they are facing and worry about what will happen in the future also when a problem has not yet manifested itself.

To our knowledge, this is the largest study investigating CSs needs in primary healthcare. The high number of participants and the inclusion of all cancer diagnosis groups are strengths of this study. Furthermore, the inclusion of both qualitative and quantitative information has revealed new insights.

The study is restricted to include those who are referred to and participate in cancer rehabilitation in primary healthcare. In principle, the CSs’ rehabilitation needs are systematically evaluated by a doctor to ensure correct referral. It has been shown that the referral process is not systematic [35], and that there are socioeconomic differences in referral and attendance to rehabilitation services [36]. If the groups that are more frequently referred (e.g. breast cancer) have specific needs (e.g. handling children’s reactions), these needs will be overestimated. Therefore, the included CSs might not be representative of all CSs in need of cancer rehabilitation.

A focus on differences in HRQOL, needs and concerns between different cancer diagnosis groups was outside the scope of this study. Nevertheless, and as indicated in Appendix 2, such differences exist. A future focus on differences between cancer diagnosis groups could contribute to the development of rehabilitation programs tailored to accommodate the specific needs of certain cancer diagnosis groups.

This study only focusses on PRO data collected at one point in a CS’s continuum of care. Future studies could include assessment at multiple points throughout the continuum of care to identify alterations in HRQOL issues and thereby guide rehabilitation interventions.

## Interpretation

This study aims to describe HRQOL, needs, and concerns among CSs referred to rehabilitation in a primary healthcare setting.

The study demonstrates that using open-ended questions adds new nuances to the understanding of CSs’ needs that cannot be extrapolated from validated scales of HRQOL. CSs report rehabilitation needs, especially in relation to functional and emotional issues, as well as physical and social/family matters. When asked openly, CSs express concerns about the uncertainty and future consequences of the disease. They worry about the impact of cancer on their children and family’s well-being, while others express a desire to maintain normality and take active steps against the disease through practices like diet and exercise.

The results emphasize the need for a holistic rehabilitation approach when addressing the complex needs of CSs outside the hospital setting.

PROs are increasingly employed in clinical practice to assess patient needs. Our findings suggest that FACT-G as a measure of HRQOL is a valuable clinical instrument for need assessment in routine rehabilitation practice. Nevertheless, open-ended questions serve as an essential supplement, as they bring forth new perspectives when directly engaging CSs.

## Supplementary Material



## Data sharing statement

Data underlying the results of this study are available from Statistics Denmark and the Health and Care Administration of the Municipality of Copenhagen. Because of restrictions of data availability, the data were used under approval from the regional ethics committee, and therefore are not publicly available.

## References

[1] Global Burden of Disease Cancer Collaboration, Fitzmaurice C, Abate D, et al. Global, regional, and national cancer incidence, mortality, years of life lost, years lived with disability, and disability-adjusted life-years for 29 cancer groups, 1990 to 2017: a systematic analysis for the global burden of disease study. JAMA Oncol. 2019;5(12):1749–68. 31560378 10.1001/jamaoncol.2019.2996 PMC6777271

[2] Miller KD, Nogueira L, Mariotto AB, et al. Cancer treatment and survivorship statistics, 2019. CA Cancer J Clin. 2019;69(5):363–85. 10.3322/caac.21565 31184787

[3] Kline RM, Arora NK, Bradley CJ, et al. Long-term survivorship care after cancer treatment – summary of a 2017 national cancer policy forum workshop. J Natl Cancer Inst. 2018;110(12):1300–10. 10.1093/jnci/djy176 30496448 PMC6658871

[4] Stein KD, Syrjala KL, Andrykowski MA. Physical and psychological long-term and late effects of cancer. Cancer. 2008;112(11 Suppl):2577–92. 10.1002/cncr.23448 18428205 PMC7047657

[5] From cancer patient to cancer survivor: lost in transition [Internet]. Washington, DC: National Academies Press; 2005 [cited 18-09-2023]. Available from: http://www.nap.edu/catalog/11468

[6] Stout NL, Santa Mina D, Lyons KD, Robb K, Silver JK. A systematic review of rehabilitation and exercise recommendations in oncology guidelines. CA Cancer J Clin. 2021;71(2):149–75. 10.3322/caac.21639 33107982 PMC7988887

[7] Kudre D, Chen Z, Richard A, et al. Multidisciplinary outpatient cancer rehabilitation can improve cancer patients’ physical and psychosocial status – a systematic review. Curr Oncol Rep. 2020;22(12):122. 10.1007/s11912-020-00979-8 33001322 PMC7529622

[8] World Health Organization. Rehabilitation [Internet]. [cited 04-01-2024]. Available from: https://www.who.int/news-room/fact-sheets/detail/rehabilitation

[9] Miroševič Š, Prins JB, Selič P, Zaletel Kragelj L, Klemenc Ketiš Z. Prevalence and factors associated with unmet needs in post-treatment cancer survivors: a systematic review. Eur J Cancer Care (Engl). 2019;28(3):e13060. 10.1111/ecc.13060 31008544

[10] Thorsen L, Gjerset GM, Loge JH, et al. Cancer patients’ needs for rehabilitation services. Acta Oncol. 2011;50(2):212–22. 10.3109/0284186X.2010.531050 21231783

[11] Lisy K, Langdon L, Piper A, Jefford M. Identifying the most prevalent unmet needs of cancer survivors in Australia: a systematic review. Asia Pac J Clin Oncol. 2019;15(5):e68–78. 10.1111/ajco.13176 31215167

[12] Licht T, Nickels A, Rumpold G, Holzner B, Riedl D. Evaluation by electronic patient-reported outcomes of cancer survivors’ needs and the efficacy of inpatient cancer rehabilitation in different tumor entities. Support Care Cancer. 2021;29(10):5853–64. 10.1007/s00520-021-06123-x 33755805 PMC8410699

[13] Harrington CB, Hansen JA, Moskowitz M, Todd BL, Feuerstein M. It’s not over when it’s over: long-term symptoms in cancer survivors—a systematic review. Int J Psychiatry Med. 2010;40(2):163–81. 10.2190/PM.40.2.c 20848873

[14] Weldring T, Smith SMS. Patient-reported outcomes (PROs) and patient-reported outcome measures (PROMs). Health Serv Insights. 2013;6:61–8. 10.4137/HSI.S11093 25114561 PMC4089835

[15] Smith TG, Castro KM, Troeschel AN, et al. The rationale for patient-reported outcomes surveillance in cancer and a reproducible method for achieving it. Cancer. 2016;122(3):344–51. 10.1002/cncr.29767 26619031

[16] Vrangbæk K. Denmark, international health care system profiles [Internet]. 2020 [cited 28-06-2023]. Available from: https://www.commonwealthfund.org/international-health-policy-center/countries/denmark

[17] Mikkelsen TB, Vind AB. Cancer rehabilitation in Denmark 2021 – rehabilitation in municipalities in the five regions of Denmark (Kommunal kræftrehabilitering i Danmark 2021. Kommunale indsatser fordelt pr. region) [Internet]. Vol. 2022. Available from: https://www.rehpa.dk/wp-content/uploads/2022/10/Kommunal-Kraeftrehabilitering-i-Danmark-2021-Kommuale-indsatser-fordelt-pr.-region.pdf [cited ited 16-02-24]

[18] Cella DF, Tulsky DS, Gray G, et al. The functional assessment of cancer therapy scale: development and validation of the general measure. J Clin Oncol. 1993;11(3):570–9. 10.1200/JCO.1993.11.3.570 8445433

[19] FACIT measures & searchable library [Internet]. [cited 19-09-2023]. Available from: https://www.facit.org/facit-measures-searchable-library

[20] Eton DT, Cella D, Yost KJ, et al. A combination of distribution- and anchor-based approaches determined minimally important differences (MIDs) for four endpoints in a breast cancer scale. J Clin Epidemiol. 2004;57(9):898–910. 10.1016/j.jclinepi.2004.01.012 15504633

[21] World Health Organisation. International Classification of Functioning, Disability and Health (ICF) [Internet]. [cited 18-09-2023]. Available from: https://www.who.int/standards/classifications/international-classification-of-functioning-disability-and-health

[22] Leonardi M, Lee H, Kostanjsek N, et al. 20 years of ICF-International Classification of Functioning, Disability and Health: uses and applications around the world. Int J Environ Res Public Health. 2022;19(18):11321. 10.3390/ijerph191811321 36141593 PMC9517056

[23] Riis-Djernæs LM, Jensen CM, Madsen E, Maribo T. Should rehabilitation goals reflect all aspects of functioning in relation to a biopsychosocial ICF perspective? Disabil Rehabil. 2021;43(12):1669–74. 10.1080/09638288.2019.1672108 31589085

[24] Cieza A, Fayed N, Bickenbach J, Prodinger B. Refinements of the ICF linking rules to strengthen their potential for establishing comparability of health information. Disabil Rehabil. 2019;41(5):574–83. 10.3109/09638288.2016.1145258 26984720

[25] Holzner B, Kemmler G, Cella D, et al. Normative data for functional assessment of cancer therapy – general scale and its use for the interpretation of quality of life scores in cancer survivors. Acta Oncol. 2004;43(2):153–60. 10.1080/02841860310023453 15163163

[26] Jabari CE, Nawajah I, Jabareen H. FACT-G assessment of the quality of life for Palestinian patients with cancer. Qatar Med J. 2022;2022(3):43. 10.5339/qmj.2022.43 36119940 PMC9475059

[27] Meiklejohn JA, Heesch KC, Janda M, Hayes SC. How people construct their experience of living with secondary lymphoedema in the context of their everyday lives in Australia. Support Care Cancer. 2013;21(2):459–66. 10.1007/s00520-012-1534-4 23010957

[28] Sterba KR, Zapka J, Gore EI, et al. Exploring dimensions of coping in advanced colorectal cancer: implications for patient-centered care. J Psychosoc Oncol. 2013;31(5):517–39. 10.1080/07347332.2013.822049 24010530

[29] LeBlanc TW, Abernethy AP. Patient-reported outcomes in cancer care—hearing the patient voice at greater volume. Nat Rev Clin Oncol. 2017;14(12):763–72. 10.1038/nrclinonc.2017.153 28975931

[30] Chen J, Ou L, Hollis SJ. A systematic review of the impact of routine collection of patient reported outcome measures on patients, providers and health organisations in an oncologic setting. BMC Health Serv Res. 2013;13:211. 10.1186/1472-6963-13-211 23758898 PMC3700832

[31] Howell D, Molloy S, Wilkinson K, et al. Patient-reported outcomes in routine cancer clinical practice: a scoping review of use, impact on health outcomes, and implementation factors. Ann Oncol. 2015;26(9):1846–58. 10.1093/annonc/mdv181 25888610

[32] Homsi J, Walsh D, Rivera N, et al. Symptom evaluation in palliative medicine: patient report vs systematic assessment. Support Care Cancer. 2006;14(5):444–53. 10.1007/s00520-005-0009-2 16402231

[33] Strömgren AS, Groenvold M, Pedersen L, Olsen AK, Sjogren P. Symptomatology of cancer patients in palliative care: content validation of self-assessment questionnaires against medical records. Eur J Cancer. 2002;38(6):788–94. 10.1016/S0959-8049(01)00470-1 11937313

[34] Ibsen C, Schiøttz-Christensen B, Maribo T, Nielsen CV, Hørder M, Handberg C. ‘Keep it simple’: perspectives of patients with low back pain on how to qualify a patient-centred consultation using patient-reported outcomes. Musculoskeletal Care. 2019;17(4):313–26. 10.1002/msc.1417 31430043

[35] Handberg C, Maribo T. Why cancer survivorship care needs assessment may lead to no clear patient pathway – based on patients’ experiences and perspectives. Eur J Oncol Nurs. 2020;48:101824. 10.1016/j.ejon.2020.101824 32942232

[36] Oksbjerg Dalton S, Halgren Olsen M, Moustsen IR, Wedell Andersen C, Vibe-Petersen J, Johansen C. Socioeconomic position, referral and attendance to rehabilitation after a cancer diagnosis: a population-based study in Copenhagen, Denmark 2010–2015. Acta Oncol. 2019;58(5):730–6. 10.1080/0284186X.2019.1582800 30905247

