# Supplementary material for: Health-related quality of life, needs, and concerns among cancer survivors referred to rehabilitation in primary healthcare setting
Source: Acta Oncol. 2024 Mar 14;63:19636. doi: 10.2340/1651-226X.2024.19636 (PMC11332470; doi:10.2340/1651-226X.2024.19636)
Supplement: Supplementary file 1 [file AO-63-19636-s1.pdf]

Supplementary material has been published as submitted. It has not been copyedited or typeset by Acta Oncologica.

Appendix 1: FACT-G mean scores individual items.

	Mean	SD
**PHYSICAL WELL-BEING**		
I lack energy*	1.8	1.1
I have nausea*	3.3	1.0
Because of my physical condition, I have trouble meeting the needs of my family*	2.5	1.2
I have pain*	2.8	1.4
I am bothered by the side effects of treatment*	2.5	1.2
I feel ill*	2.7	1.1
I am forced to spend time in bed*	3.3	1.0
**SOCIAL/FAMILY WELL-BEING**		
I feel close to my friends	3.0	1.1
I get emotional support from my family	3.4	0.9
I get support from my friends	3.0	1.1
My family has accepted my illness	3.0	1.0
I am satisfied with family communication about my illness	3.2	1.0
I feel close to my partner (or the person who is my main support)	3.3	1.1

I am satisfied with my sex life	1.4	1.4
**EMOTIONAL WELL-BEING**		
I feel sad*	2.5	1.1
I am satisfied with how I am coping with my illness	2.6	1.1
I am losing hope in the fight against my illness*	3.7	0.7
I feel nervous*	2.7	1.1
I worry about dying*	2.9	1.1
I worry that my condition will get worse*	2.2	1.2
**FUNCTIONAL WELL-BEING**		
I am able to work (include work at home)	1.8	1.1
My work (include work at home) is fulfilling	1.8	1.2
I am able to enjoy life	2.2	1.0
I have accepted my illness	2.4	1.2
I am sleeping well	2.2	1.2
I am enjoying the things I usually do for fun	2.1	1.2
I am content with the quality of my life right now	1.8	1.2

**Item scores are reversed when calculating item mean score for negatively stated-items*

Appendix 2: *Mean total FACT-G scores and subscale scores for different cancer diagnosis groups*

	% (n)	Physical well-being (SD)	Emotional well-being (SD)	Functional well-being (SD)	Social well-being (SD)	Total FACT-G (SD)
**Score range**		0-28	0-28	0-28	0-24	0-108
**Total sample**	100 % (2,643)	18.9	16.6	14.4	21.1	71.1
**Cancer diagnosis**						
Mesothelial and soft tissue (C45-C49)	1.0 % (26)	16.4 (4.2)	15.0 (5.4)	13.2 (5.0)	21.5 (3.9)	66.3 (13.2)
Lymphoid tissue (C81-CC90)	7.5 % (173)	17.1 (5.5)	16.6 (4.0)	12.9 (5.0)	20.2 (5.2)	66.8 (14.0)
Female genital organs (C51-C58)	5.5 % (145)	17.3 (6.2)	15.9 (4.8)	13.6 (5.5)	20.4 (5.7)	67.3 (16.9)
Digestive organs (C15-C26)	16.6 % (439)	17.9 (5.5)	16.2 (5.0)	13.1 (5.8)	20.7 (5.3)	67.9 (16.2)
Unknown	5.1% (134)	18.0 (5.7)	16.6 (4.8)	14.1 (6.1)	20.1 (5.6)	68.8 (17.3)
Ill-defined, secondary, and unspecified sites (C76-C80)	2.5 % (65)	19.1 (5.5)	15.8 (4.3)	14.0 (5.4)	20.2 (5.5)	69.1 (15.9)

Lip, oral cavity, and pharynx (C00-14)	5.4 % (143)	17.3 (5.6)	17.3 (4.6)	13.4 (5.9)	21.5 (4.7)	69.6 (15.7)
Respiratory and intrathoracic organs (C30-C39)	10.3 % (273)	18.4 (5.5)	16.5 (4.7)	13.7 (5.6)	21.1 (5.5)	69.9 (16.6)
Melanoma and other malignant neoplasms of skin (C43-C44)	1.4 % (37)	19.2 (6.1)	15.3 (4.9)	14.9 (6.7)	20.6 (4.2)	70.3 (17.0)
Urinary tract (C64-C68)	2.3 % (60)	18.4 (5.9)	16.8 (4.5)	14.8 (5.7)	21.6 (5.1)	71.7 (15.6)
Male genital organs (C60-C63)	7.5% (197)	19.8 (5.1)	17.3 (4.3)	15.5 (5.5)	20.6 (4.6)	73.1 (14.8)
Haematopoietic tissue (C91-C96)	2.2 % (59)	18.2 (5.9)	18.0 (4.6)	16.0 (5.1)	21.8 (5.2)	74.0 (15.3)
Breast (C50)	32.1 % (848)	20.5 (5.0)	16.9 (4.5)	15.4 (5.5)	21.8 (5.2)	74.6 (15.2)
Eye, brain, and other parts of central nervous system (C69-C72)	1.7 % (44)	21.0 (4.2)	16.4 (4.2)	16.7 (4.9)	21.2 (4.8)	75.1 (12.9)
